# Sensitive and Extraction-Free Detection of Methicillin-Resistant *Staphylococcus aureus* through Ag^+^ Aptamer-Based Color Reaction

**DOI:** 10.4014/jmb.2308.08044

**Published:** 2023-09-28

**Authors:** Hongli Cao, Guosheng Zhang, Hui Ma, Zhongwen Xue, Ran Huo, Kun Wang, Zijin Liu

**Affiliations:** 1 Emergency Department, Beijing Rehabilitation Hospital, Capital Medical University, Beijing 100144, P.R. China; 2 Orthopedic Rehabilitation Department, Beijing Rehabilitation Hospital, Capital Medical University, Beijing 100144, P.R. China

**Keywords:** Methicillin-resistant *Staphylococcus aureus*, silver ion, exonuclease-III, PBP2a aptamer

## Abstract

Refractory infections, such as hospital-acquired pneumonia, can be better diagnosed with the assistance of precise methicillin-resistant *Staphylococcus aureus* (MRSA) testing. However, traditional methods necessitate high-tech tools, rigorous temperature cycling, and the extraction of genetic material from MRSA cells. Herein, we propose a sensitive, specific, and extraction-free strategy for MRSA detection by integrating allosteric probe-based target recognition and exonuclease-III (Exo-III)-enhanced color reaction. The penicillin-binding protein 2a (PBP2a) aptamer in the allosteric probe binds with MRSA to convert protein signals to nucleic acid signals. This is followed by the DNA polymerase-assisted target recycle and the production of numerous single-strand DNA (ssDNA) chains which bind with silver ion (Ag^+^) aptamer to form a blunt terminus that can be identified by Exo-III. As a result, the Ag^+^ aptamer pre-coupled to magnetic nanoparticles is digested. After magnetic separation, the Ag^+^ in liquid supernatant catalyzes 3,3’,5,5’-tetramethylbenzidine (TMB) for a color reaction. In addition, a concentration of 54 cfu/mL is predicted to be the lowest detectable value. Based on this, our assay has a wide linear detection range, covering 5 orders of magnitude and demonstrating a high specificity, which allows it to accurately distinguish the target MRSA from other microorganisms.

## Introduction

Drug-resistant microorganisms are a major health concern since they significantly restrict available treatment choices [1-3]. Nosocomial infections, such as pneumonia, have a greater mortality rate than other types of hospital-acquired illnesses and hence require extensive antibiotic therapy [4, 5]. Methicillin-resistant *Staphylococcus aureus* (MRSA) is a group of antibiotic-resistant bacteria that cause nosocomial infections such as hospital-acquired pneumonia [6]. MRSA is resistant to the majority of antibiotics in use, and it is responsible for a wide variety of medical issues in addition to a high mortality rate [7]. For this reason, accurate and rapid detection of MRSA strains is fundamental for guiding appropriate patient treatment in the early stages of infections and preventing the spread of MRSA.

Traditional antimicrobial susceptibility testing is considered the gold standard for MRSA identification because of its ease of use; nevertheless, its lengthy delay and potential for false-negative results limit its practicality [8-10]. Although it is highly specific, the high cost of the antibody prevents it from being widely used in immunological research. Emerging methodologies based on nucleic acids appear to be superior to other methods since they are capable of directly identifying the related antibiotic resistance genes. The sensitivity of established and developing isothermal amplification techniques, such as loop-mediated isothermal amplification and nucleic acid sequence-based amplification, is commensurate with that of the general polymerase chain reaction (PCR) [11-14]. For instance, Luyu Wei *et al*. presented a CRISPR (clustered regularly interspaced palindromic repeats)/Cas12a (CRISPR associated nucleases 12a) system with recombinase polymerase amplification (RPA) for the colorimetric detection of MRSA [15]. MRSA was detected at concentrations as low as 8 CFU/ml using the triple amplification of RPA and the multi-turnover nuclease activity of Cas12a. Using a CRISPR-associated protein 9/single-guide RNA (dCas9/sgRNA) combination as a targeting material and SYBR Green I (a fluorescent dye that, when attached to double-stranded DNA (dsDNA), emits a bright green fluorescence) as a fluorescent probe, Kyeonghye Guk *et al*. showed a CRISPR-mediated DNA-FISH approach for the easy, fast, and highly sensitive detection of MRSA [16]. These techniques, however, necessitate high-tech tools and rigorous temperature cycling. In addition, the detection results may be influenced because these techniques call for the extraction of genetic material from MRSA cells. Therefore, the development of a sensitive, specific and extraction-free strategy for MRSA detection is crucial.

An aptamer is a small oligonucleotide molecule that binds selectively to a protein. Aptamers have been utilized in the development of biosensors due to their high protein affinities [17, 18]. Penicillin-binding protein 2a (PBP2a) is encoded by the *mecA* gene in MRSA, which confers resistance [19-21]. In the presence of antibiotics, the protein catalyzes a transpeptidation event necessary for cell wall production and peptidoglycan cross-linking. The utilization of PBP2a aptamer has made it possible to detect MRSA without extraction of DNA sequences.

In this work, a colorimetric biosensor based on an allosteric probe was created for the ultrafast, ultrasensitive, and extraction-free detection of MRSA by use of the exonuclease-III (Exo-III)-augmented Ag^+^ aptamer-based color reaction. The advantages of the established method include: (i) high specific target recognition because the PBP2a aptamer is integrated in the allosteric probe; (ii) DNA polymerase cooperating with endonuclease to construct multiple signal amplification, endowing the method with a high sensitivity; and (iii) the method does not require DNA extraction. For the reasons stated above, this method holds great potential as a means for identifying MRSA.

## Materials and Methods

### Materials and Instrumentation

All reagents and chemicals were of the highest quality for analytical application. The American Type Culture Collection (ATCC) was consulted for strains of *Pseudomonas aeruginosa* (*P. aeruginosa*), *Escherichia coli* (*E. coli*), *Bacillus subtilis* (*B. subtilis*), *Bacillus coagulans* (*B. coagulans*), and methicillin-susceptible *Staphylococcus aureus*. Thermo Scientific (USA) supplied the Bsm DNA polymerase and the Nb. Bpu10I nicking endonuclease. Deoxyribonucleoside triphosphates (dNTPs) were procured from TaKaRa Bio Inc. (China). The DNA sequences listed in Table S1 were synthesized and HPLC-purified by Sangon Biotech Co., Ltd. (China).

### Bacterial Strains and Cultivation Conditions

All strains of *P. aeruginosa*, *E. coli*, and *B. subtilis* were stored in our facility for future use. The strains were grown in LB medium at 37°C with an agitation rate of 220 rpm for 24 h. The bacteria were harvested by centrifugation at 3,000 ×*g* for 5 min, and then washed gently in 10 mM PBS (including 145 mM NaCl, pH 7.4) to get rid of any leftover media. The total number of bacteria in the LB medium was determined by serially diluting the bacterial suspension. The plate count agar was then incubated at 37°C for 24 h with repeated dilutions of 100 ml. The amount of viable bacteria was calculated from the number of plate-grown colonies and expressed as colony-forming units per milliliter (CFU/ml).

### MRSA Detection

Both the allosteric probe and the H probe were preheated to 95°C for 5 min before being cooled to 37°C to create their respective secondary structures. A total of 80 ml of a 10 mM PBS buffer (including 145 mM NaCl, pH 7.4) was added to 100 ml of MRSA samples at varying concentrations. The mixtures were heated to 37°C and stirred at 220 rpm for 15 min. Following incubation at 37°C for 25 min, we added 250 mM dNTPs, 0.2 U/ml Nb. Bpu10I, 0.15 U/ml Bsm DNA polymerase, and 10 mM MB-Ag^+^-aptamer. Then, 25 ml of 5 mM AgNO_3_ was added, and the mixture was incubated at 37°C for 10 min after being subjected to magnetic separation and two washes in ultrapure water. After that, we added 35 ml of 5 mM TMB and let it react at room temperature in the dark for 25 min. Finally, absorbance at 652 nm was measured, and the results were in agreement with that visible to the naked eye.

## Results and Discussion

### Design Rationale of the Colorimetric Approach

**Fig. 1:**
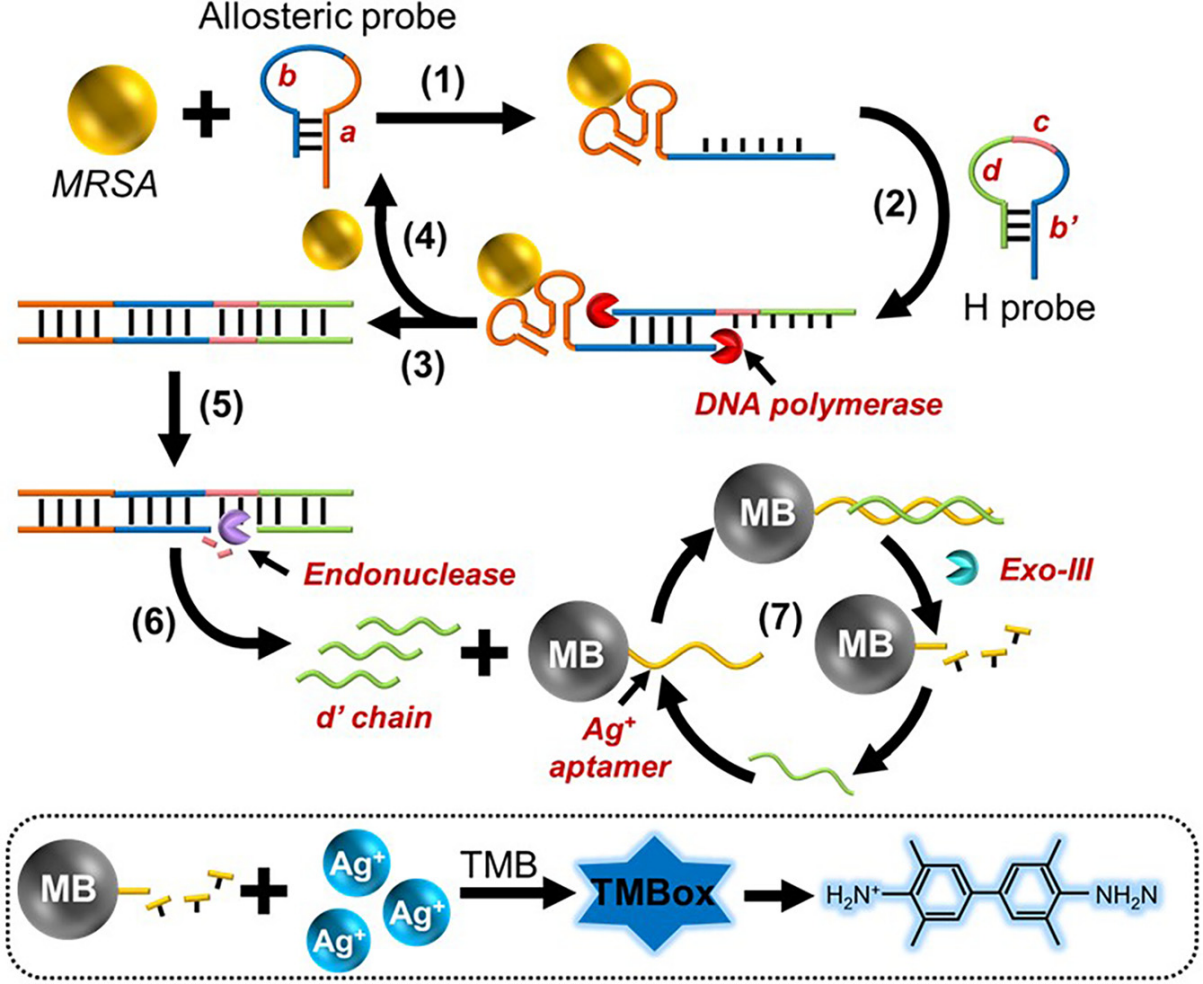
The working rationale of the allosteric probe-based approach. The method includes the signal amplification process (steps 1-7) and the color reaction (dotted square).

The detection principle is shown in Fig. 1. In our method, an allosteric probe with a hairpin structure was designed. There are in total two functional sections in the allosteric probe, including the “a” section and “b” section. The “a” section is PBP2a aptamer and the “b” section is capable of initiating the amplification process. The PBP2a protein aptamer is a small oligonucleotide molecule that binds selectively to a PBP2a protein which is encoded by the *mecA* gene in MRSA. When MRSA exists in the sensing system, “a” section binds with PBP2a protein, and as a result, the “b” section is exposed. The “b” section unfolds H probe to release the “c” and “d” sections in the H probe. With the “b” section as primer, the chain is extended from the 5’ terminus to the 3’ terminus under the assistance of DNA polymerase, in which process the aptamer sequence is occupied and MRSA is released from the aptamer. The released MRSA binds with an additional allosteric probe to form a signal cycle. With the “b” section as primer, a chain that is complementary with “c” and “d” section is produced. The “c” section can be identified by endonuclease to generate a nicking site. By cooperating with the DNA polymerase, numerous “d” chains are generated. The “d” chains can bind with Ag^+^ aptamers to form a blunt 3’ terminus that can be recognized by Exo-III. As a result, the Ag^+^ aptamers that are originally fixed on the surface of magnetic bead (MB) are digested. By magnetic separation, only MB would remain in the sediment. Additionally, the organic dye 3,3',5,5'-tetramethylbenzidine (TMB), which may be oxidized by Ag^+^, was utilized as a visualization probe. Therefore, Ag^+^ was unable to attach to the aptamer and instead interacted with TMB to produce a color reaction. In this instance, the solution containing oxidized TMB (TMBox) displayed a blue hue and a UV–vis absorption peak at 652 nm.

### Feasibility of Allosteric Probe and Color Reaction

**Fig. 2:**
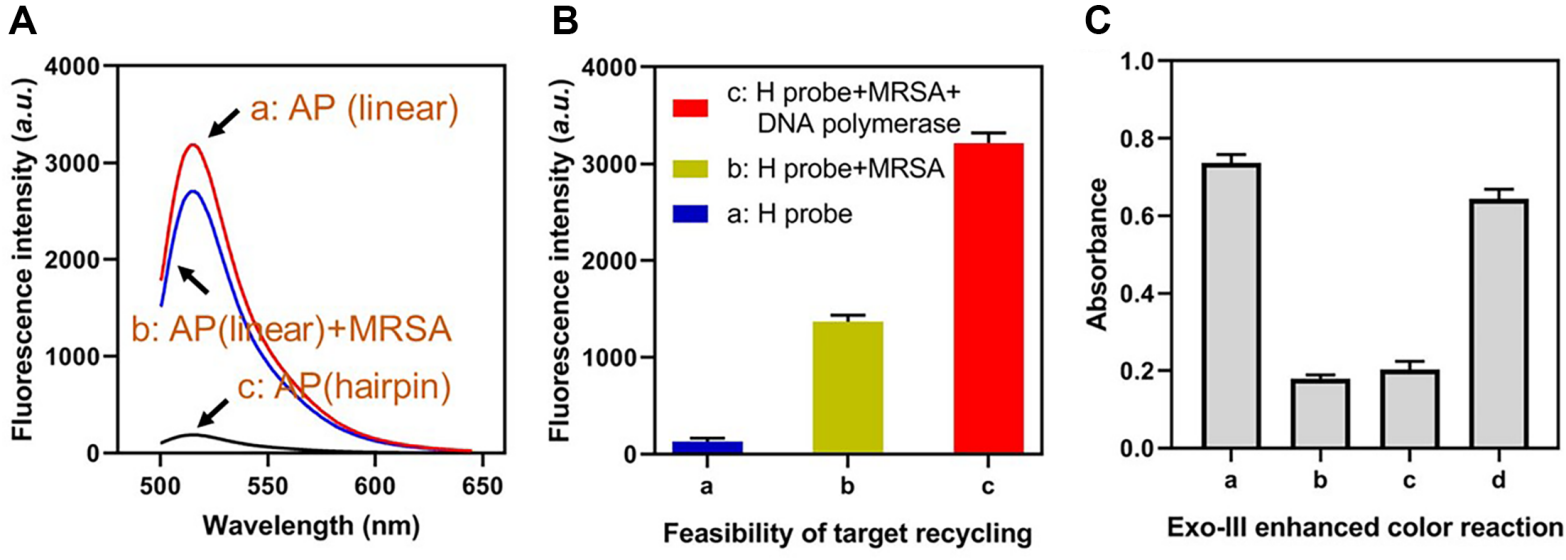
Construction of the allosteric probe and feasibility of Exo-III-enhanced color reaction. (**A**) Fluorescent spectrum of FAM-labeled allosteric probe before and after assembly. (**B**) Fluorescent intensity of the FAM-labeled H probe in the target recycling process. (**C**) Absorbance of the Exo-III-enhanced color reaction under different experimental conditions.

The assembly of an allosteric probe and its viability in identifying MRSA were evaluated using a fluorescent assay. In this fluorescent assay, the allosteric probe was labeled at its 5’-terminus with a FAM fluorophore (carboxyfluorescein) and its 3’-terminus with a DABCYL quenching group (4-dimethylamino-azobenze-4’-carboxylic acid). Due to the close proximity between FAM and DABCYL in the hairpin structure, the fluorescence signal of the allosteric probe is dampened. Fig. 2A demonstrates the altered fluorescent intensities of allosteric probes with distinct conformations. Fluorescence intensity in the linear state was nearly 7.54 times greater than that of the hairpin structure, indicating the successful assembly of the allosteric probe. The recovery of fluorescence intensity upon addition of MRSA indicates that the hairpin structure of the allosteric probe was successfully dissociated. In addition, the two termini of the H probe were labeled with FAM fluorophore and DABCYL quenching group in order to test the target cycle. When MRSA was present in the sensing system, a significantly augmented fluorescence signal was detected, indicating that the H probe was unfolded. Upon addition of DNA polymerase, the system fluorescence intensity increased by 2.32-fold compared to the absence of DNA polymerase, indicating the formation of a target cycle (Fig. 2B). Using synthesized d' chain, we then investigated the viability of the Exo-III-enhanced Ag+ aptamer-based color reaction. As shown in Fig. 2C, the absorbance was low in the absence of “d” chain, Exo-III, and TMB in the sensing system. Only when all of these components were present did the absorbance significantly increase. The pigment change corresponded to the absorbency.

### Optimization of the Detection Conditions

**Fig. 3:**
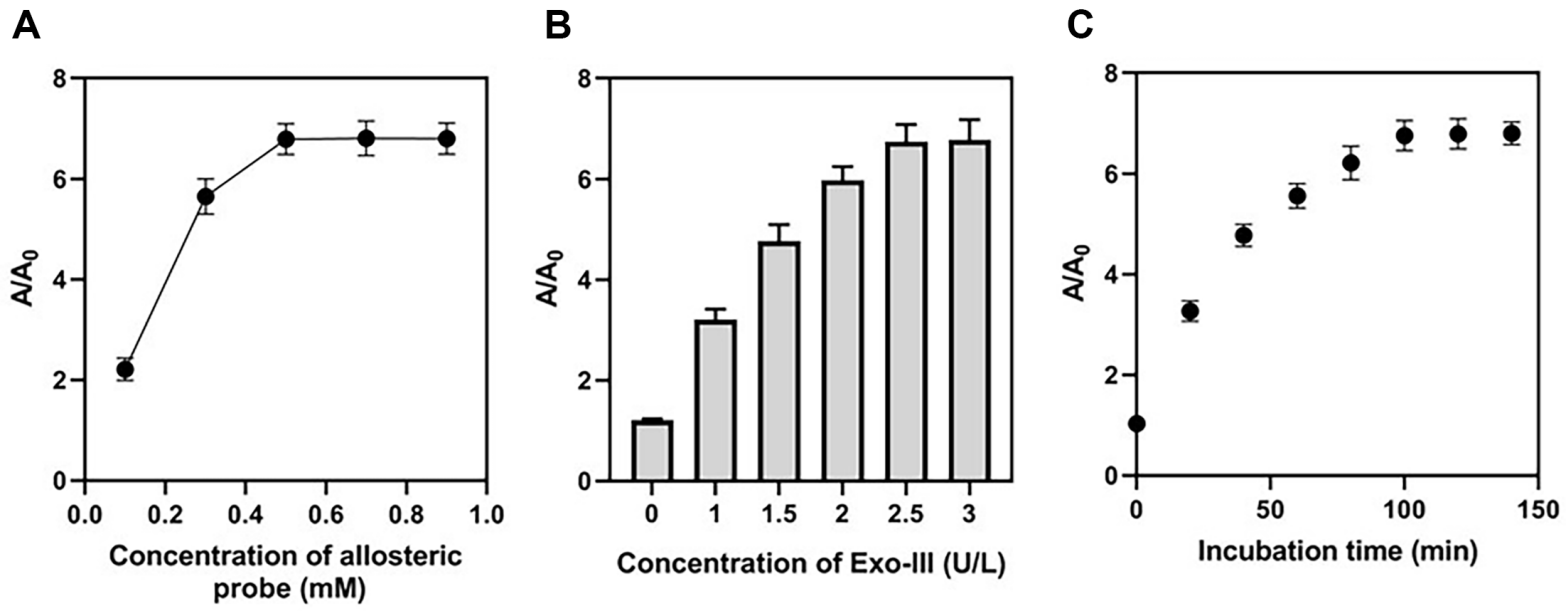
Optimization of experimental conditions. Shown above are the effects of (**A**) the concentration of allosteric probe, (**B**) the concentration of Exo-III, and (**C**) the incubation period on the MRSA detection performance.

To maximize the overall performance of the colorimetric approach, it was necessary to optimize a number of different experimental settings. The absorbance ratio of the solution with and without MRSA, denoted by A/A0, was employed as an indication to assess the experimental settings during the optimization process. The effects of allosteric probe concentration, Exo-III concentration, and incubation time on the value of A/A0 were investigated. In identifying MRSA, the concentration of the allosteric probe is essential. Figure 3A demonstrates that as the initial allosteric probe concentrations are raised, the A/A0 ratio improves. The highest value of A/A0 was seen when an allosteric probe of 0.5 mM was employed in the experiment. The optimum concentration of the allosteric probe was determined to be 0.5 mM. Increasing the Exo-III concentration in the testing solution can result in a more pronounced response in the Exo-III-enhanced color reaction. As seen in Fig. 3B, the absorbance rises as the Exo-III concentration increases. The optimum Exo-III concentration was determined to be 2.5 U/L since this value corresponds to the point at which the A/A0 becomes saturated. Moreover, investigation of the incubation period showed that the right duration can guarantee the expected color reaction and speed up the total detection process. As Fig. 3C shows, the ratio A/A0 rises as incubation time increases, peaking at 100 min. Therefore, a 100-min incubation period was used for the detection.

### Performance of the Approach

**Fig. 4:**
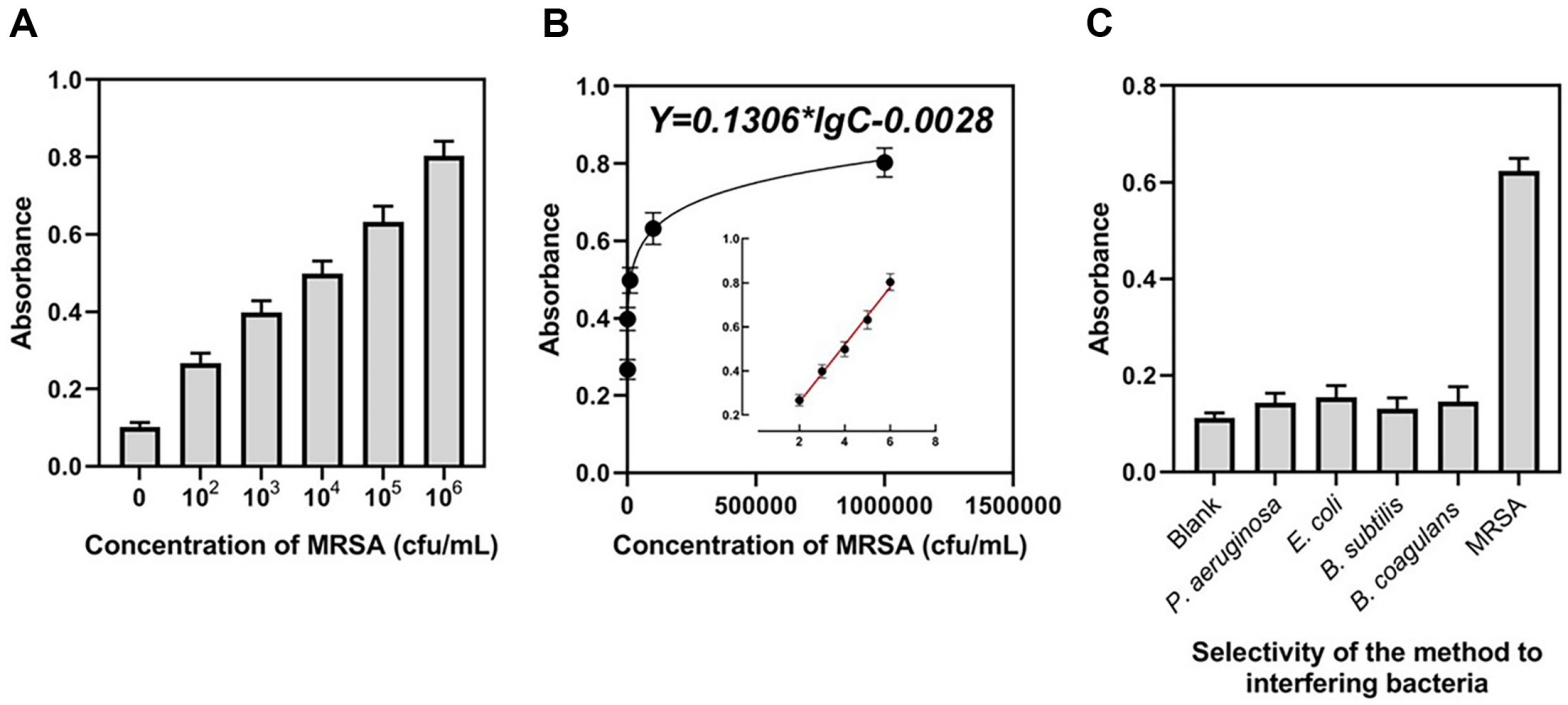
Analytical performance of the approach in detecting MRSA. (**A**) Absorbance of the approach when detecting different concentrations of MRSA. (**B**) Linear correlation between the absorbance and concentrations of MRSA. (**C**) Absorbance of the approach when detecting different bacteria of the same concentration.

The sensitivity and dynamic range were tested under ideal experimental settings. The samples consisted of MRSA suspensions that had been diluted serially. Figure 4A shows that as the MRSA concentration is raised from 10^2^ to 10^6^ CFU/ml, the absorbance also increases. MRSA concentration is proportional to the logarithm of the absorbance, which is a linear relationship. As shown in Fig. 4B, the linear regression equation between absorbance (Y) and MRSA concentration (C) is Y = 0.1306*lgC - 0.0028 (R^2^, 0.9935). A concentration of 54 CFU/ml (3 standard deviations of the blank signal/sensitivity) is predicted to be the lowest detectable value, which is superior or comparable to former methods (Table S2). Thus, the absorbance offers a simple and highly sensitive approach for detecting MRSA across a wide concentration range of more than five orders of magnitude. The present colorimetric detection method has been demonstrated to be highly specific through testing conducted on numerous non-target bacteria, such as *P. aeruginosa*, *E. coli*, *B. subtilis*, and *B. coagulans*. As a countermeasure, we also included a sample that lacked any bacteria. Compared to the target MRSA, the absorbance of the samples containing other bacteria and the control is substantially lower (Fig. 4C), thereby indicating a high degree of target specificity in the experiment.

### Detection of MRSA in Commercial Serum Samples

**Fig. 5:**
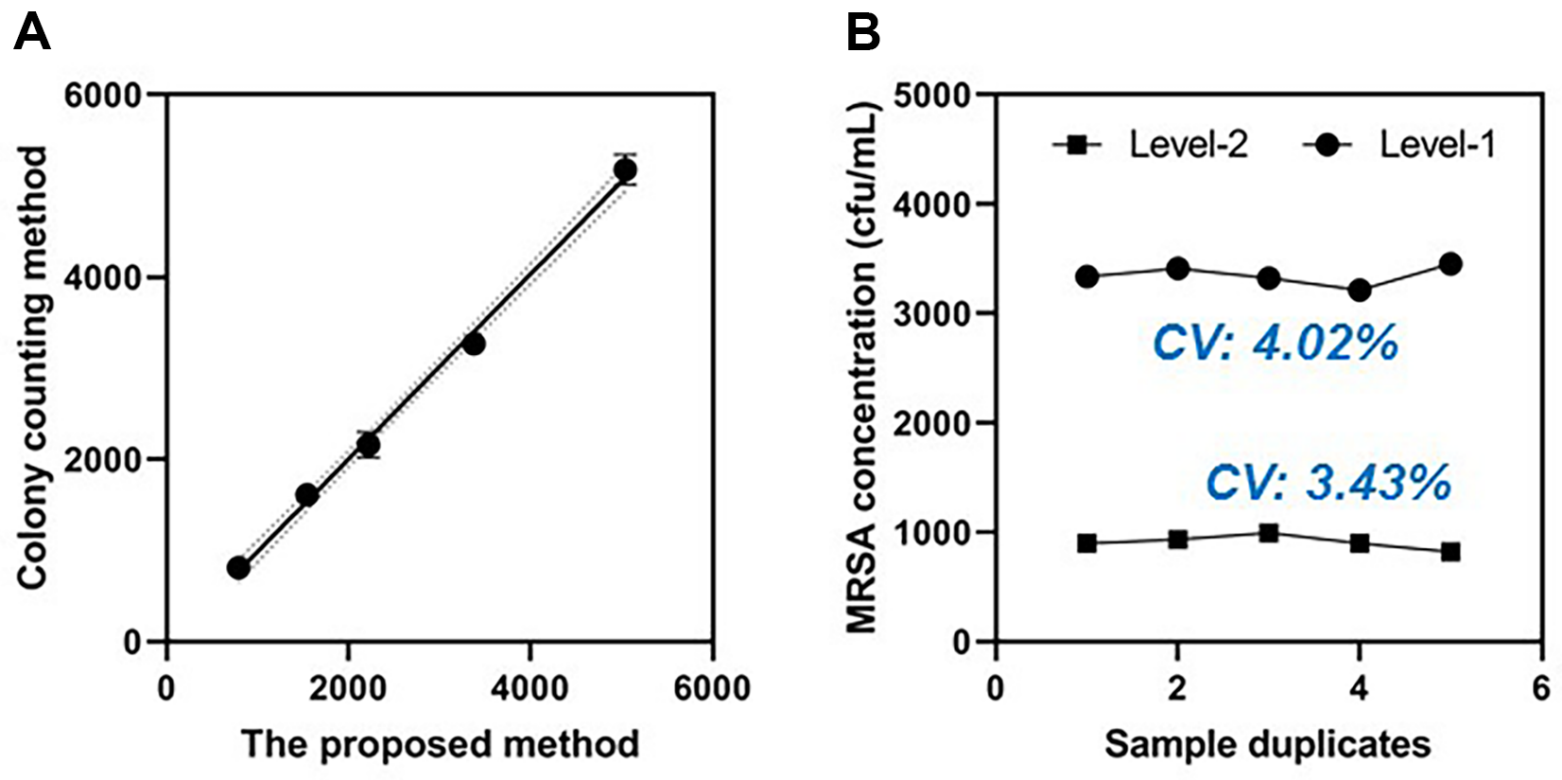
Clinical application of the approach. (**A**) Correlation between the calculated MRSA concentration by the method and traditional colony-counting method. (**B**) MRSA concentrations of high level (level-2) and low level (level-2) detected by the method.

Detection of MRSA in clinical samples is important in infection monitoring. To verify the practicability of the proposed detection, commercial serum samples were selected to prepare clinical samples for the quantitative detection of MRSA without pretreatment. To prepare clinical samples, the cultivated bacteria were spiked into the serum sample to reach different final concentrations. The results shown in Fig. 5A reveal the favorable correlation between the calculated MRSA amounts by our method and the traditional culture method. In addition, 10 sample duplicates were detected by the method to test the repeatability of the approach. As shown in Fig 5B, the variable coefficient of the 10 samples’ detection was 3.43% and 4.02%, which indicates the sufficient potential of the approach in clinical application.

## Conclusion

In this work, we developed an allosteric probe-based colorimetric biosensor that uses the Exo-III-improved Ag^+^ aptamer-based color reaction to quickly, ultra-sensitively, and without extraction detect MRSA. In the presence of its target, the PBP2a protein aptamer sequence in an allosteric probe causes a conformational change that sets off a cascade of downstream signaling events. When Ag^+^ aptamers are cleaved using the Exo-III-assisted recycling procedure, the color reaction is significantly amplified. A concentration of 54 CFU/ml is predicted to be the lowest detectable value. The assay has a wide linear detection range, covering 5 orders of magnitude. In addition, the assay demonstrates a high specificity, allowing it to accurately distinguish the target MRSA from other microorganisms within 100 min. In addition, we also used the assay to successfully detect MRSA in fake clinical samples, indicating its potential use in the diagnosis of pneumonia and other serious infections.

## Supplemental Materials

Supplementary data for this paper are available on-line only at http://jmb.or.kr.
