# Supplementary material for: Sensitive and Extraction-Free Detection of Methicillin-Resistant Staphylococcus aureus through Ag+ Aptamer-Based Color Reaction
Source: J Microbiol Biotechnol. 2023 Sep 28;34(1):192–7. doi: 10.4014/jmb.2308.08044 (PMC10840478; doi:10.4014/jmb.2308.08044)
Supplement: Supplementary file 1 [file jmb-34-1-192-supple.pdf]

J. Microbiol. Biotechnol.
https://doi.org/10.4014/jmb.2308.08044

Supplementary Tables

Table S1. Sequences of oligonucleotides used in this work.

Title	Sequences (5' to 3')
Ag^+ aptamer	CCT CCC TCC CTC CCT TTT TCC CAC CCA CCC ACC
d' chain	GGT GGG TGG CTC GGA AAA
H probe	CAC CCC ACC TCG TAT TTT TT CC TAA GCC CTC CCT CCC TCC CTT TTT CCC ACC AAA ATA CGA GGT
Allosteric probe	CAC CCC ACC TCG CTC CCG TGA CAC TAA TGC TAT TTT TTT CAC CCA CCG AGG TGG GGT G

Table S2. A brief comparison of the proposed method with former ones.

Title	Principle	LOD	Target	Signal mode	Incubation time	Extraction	Ref.
The method	Exo-III assisted signal amplification	54 cfu/mL	Bacteria	Color	100 min	-	
Functional chimera method	SDA reaction	80 cfu/mL	Bacteria	Fluorescence	80 min	-	[1]
CRISPR-Cas12a	RCA	100 cfu/mL	Bacteria	Fluorescence	80 min	-	[2]
Fluorescence biosensor	Exo-III assisted cascade signal amplification strategy	2.4 fM	*mecA* gene	Fluorescence	More than 100 min	+	[3]
Low-cost colorimetric diagnostic screening assay	Antibody-antigen reaction	10^3 cfu/mL	Gene analysis	Color	Not mentioned	+	[4]

SDA, strand displacement amplification; RCA, rolling circle amplification; Exo-III, exonuclease-III;

Ref

1. Cai R, Yin F, Zhang Z, Tian Y, Zhou N. 2019. Functional chimera aptamer and molecular beacon based fluorescent detection of Staphylococcus aureus with strand displacement-target recycling amplification. *Anal Chim Acta.* **1075:** 128-136.
2. Xu L, Dai Q, Shi Z, Liu X, Gao L, Wang Z*, et al.* 2020. Accurate MRSA identification through dual-functional aptamer and CRISPR-Cas12a assisted rolling circle amplification. *J Microbiol Methods.* **173:** 105917.
3. Li Q, Zhou D, Pan J, Liu Z, Chen J. 2018. Ultrasensitive and simple fluorescence biosensor for detection of the mecA gene of Staphylococcus aureus by using an exonuclease III-assisted cascade signal amplification strategy. *Analyst.* **143:** 5670-5675.
4. Raji MA, Chinnappan R, Shibl A, Suaifan G, Weber K, Cialla-May D*, et al.* 2021. Low-cost colorimetric diagnostic screening assay for methicillin resistant Staphylococcus aureus. *Talanta.* **225:** 121946.
